# Epileptiform Neuralgia

**Published:** 1877-09-20

**Authors:** 


Epileptiform Neuralgia. — In the Mobile Medical Society was reported a case, under the care of Dr. Cochran, of a lady, fifty years of age, affected with epileptiform neuralgia. All the anti-neuralgic remedies had been used, as well as quinine, nux vomica, electricity (faradic current), bromide of potassium, and hydrate of chloral. The continuous current, cod-liver oil, and phosphorus, in the hands of another physician, relieved her for a time. It was only for a time, however, when she was again worse. Dr. Cochran determined to try Trousseau's plan. This consists in "commencing with moderate doses of opium, and gradually increasing the amount given until the desired effect is accomplished." Twenty drops of chlorodine were ordered morning and evening. She improved rapidly. He continued the drug in gradually decreasing doses for a time. She continues well, and shows no sign of a relapse. Just how long a period of freedom from the disease the Doctor does not state.

